# Optimum Energy Management for Air Conditioners in IoT-Enabled Smart Home

**DOI:** 10.3390/s22197102

**Published:** 2022-09-20

**Authors:** Ashleigh Philip, Shama Naz Islam, Nicholas Phillips, Adnan Anwar

**Affiliations:** 1 Deakin University, Waurn Ponds, VIC 3216, Australia; 2 Itron Australasia, Melbourne, VIC 3000, Australia; 3 Strategic Centre for Cyber Security Research and Innovation (CSRI), School of Information Technology, Deakin University, Waurn Ponds, Geelong, VIC 3216, Australia

**Keywords:** energy management, pre-cooling, air conditioners, IoT-enabled smart home, time of use

## Abstract

This paper addresses the optimal pre-cooling problem for air conditioners (AC) used in Internet of Things (IoT)-enabled smart homes while ensuring that user-defined thermal comfort can be achieved. The proposed strategy utilises renewable energy generation periods and moves some of the air conditioning loads to these periods to reduce the electricity demand. In particular, we propose a multi-stage approach which maximises the utilisation of renewable energy at the first stage to satisfy air conditioning loads, and then schedules residual energy consumption of these loads to low price periods at the second stage. The proposed approach is investigated for the temperature and renewable generation data of NSW, Australia, over the period 2012–2013. It is shown that the approach developed can significantly reduce the energy consumption and cost associated with AC operation for nearly all days in summer when cooling is required. Specifically, the proposed approach was found to achieve a 24% cost saving in comparison to the no pre-cooling case for the highest average temperature day in January, 2013. The analysis also demonstrated that the proposed scheme performed better when the thermal insulation levels in the smart home are higher. However, the optimal pre-cooling scheme can still achieve reduced energy costs under lower thermal insulation conditions compared to the no pre-cooling case.

## 1. Introduction

There is currently a global energy crisis, as energy consumption across the world is increasing at a significant rate every year. Residential consumers contribute significantly to this total global energy consumption, to seasonal and daily peak demands, and account for a total of 30% to 40% of energy consumption around the world [1]. A significant proportion of residential energy consumption is attributed to heating, cooling, ventilation and air conditioning (HVAC) systems. For example, in Finland, HVAC loads from the residential sector account for 70% of total annual energy consumption [2]. On the other hand, 40% of the residential energy consumption in Australia comes from space conditioning [3]. As renewable generation, smart-grid technologies and smart meters are being added into the network, there is a renewed interest in integrating demand-side management strategies into the network with decreasing technological barriers [4,5]. The advancement of Internet of Things (IoT) technologies has allowed the integration of intelligent algorithms for demand management applications in smart homes. Residential demand management tools, such as home energy management systems (HEMS), can assist in significantly reducing peak load demand by integrating optimum operational strategies for energy consumption in smart homes [6]. Thus, effective demand management schemes need to be integrated in residential HEMSs to reduce the energy consumption of HVAC systems [7].

Existing demand response initiatives rely on incentive-based programs where participants are offered participation credits, or credits based on performance. These programs include direct load control (DLC) [8], interruptible/curtailable (I/C) load [9], demand bidding (DB) [10] and ancillary service market (ASM) programs [11]. In addition, there are time-based programs which use time-varying prices based on the system load. Customers receive the price information and can choose to lower their consumption when rates are high. There are several pricing methods, including time-of-use (ToU) [12], critical-peak pricing (CPP) [13], peak-load pricing (PLP) [14], and real-time pricing (RTP) [15]. These demand response strategies can be either centralised [16] or decentralised [17]. The design of an efficient DR program for residential consumers is considered more complicated compared than that for industrial consumers mainly due to the near-random consumption patterns that require more complex modelling [18]. One of the obstacles to the adoption of residential demand response is the lack of customers’ knowledge in responding to time-varying prices and incentives. As a result, automated control systems for generating appropriate response to these signals offer a viable solution for residential demand management. A number of such techniques have been proposed for controllable appliances and thermostatically controlled loads (TCLs), where the goal of HEMS is to minimize customer costs, while still maintaining the pre-defined operational constraints set by the user [19]. Controllable devices, such as cooling and heating systems, can have maximum and minimum range limits defined by the customer for every time step. Additionally, HEMS will receive input information, such as pricing data, to consider to optimise the operation of TCLs [20]. Existing research on demand management problems often focuses on designing optimum energy management strategies for HEMSs. In the following sub-section, related studies on optimum energy management for demand response through load control are described.

### 1.1. Related Works

In existing research, for effective residential demand management, the trade-off between the cost of energy and the inconvenience cost has been considered as a critical factor for the encouragement of customer commitment and satisfaction with demand response programs. If a customer has a higher budget, they may be less likely to be tolerant of inconvenience or discomfort with higher energy costs. There are several approaches that have been described in the literature to model customer preferences and convenience as an objective function, either considering these as a set of constraints, or attempting to model energy costs simultaneously with consumer inconvenience. For example, the authors in [21] considered minimising the difference between preferred and optimal schedules using non-linear quadratic programming. However, the use of non-linear models may increase complexity which could affect the ability of the HEMS to provide an optimal solution in a reasonable time. A multi-objective optimisation problem, to achieve a flat demand profile while minimising energy costs through appliance scheduling, was represented in [22] as a non-convex mixed integer programming problem. Other approaches have used mixed-integer linear programming (MILP) models to optimise the electricity cost, while also maintaining technical operation constraints and consumer preferences [1]. However, these approaches are not sufficiently scalable to handle a large number of variables.

Recently, a number of meta-heuristic optimisation algorithms, such as grey wolf optimisation (GWO) [23], teaching/learning-based optimisation (TLBO) [24], the whale optimisation algorithm (WOA) [25], Harris hawks optimisation (HHO) [26], moth flame optimisation (MFO) [27] and sine cosine optimisation (SCO) [28] have been proposed in the context of energy management systems. The authors of [29] considered GWO to optimise energy management, as well as battery sizing for grid-connected microgrids, which improved renewable energy utilisation and reduced electricity cost. GWO has been integrated with the energyplus simulation tool developed by the U.S. Department of Energy to improve building energy optimisation in [30], considering the thermal performance of the building through the built-in functionality of energyplus. The authors of [31] adopted an inertia-weight local search algorithm with TLBO to optimise the energy management problem in an islanded microgrid with renewable energy sources. On the other hand, WOA was used to optimise energy management in a microgird integrated with renewable resources and demand response programs in [32]. However, the two research papers did not consider load types and how to optimise the consumption of individual loads. The authors of [33] utilised WOA to optimise an artificial neural network model which was able to predict the heating/cooling load of a building with greater accuracy, but there was no consideration of how this load could be controlled. An optimum appliance scheduling problem was solved in [26] using HHO which sought to improve electricity costs as well as the peak-to-average ratio (PAR) for electricity demand, while reducing wait time for users to use their controllable appliances. However, the authors did not consider controlling heating/cooling loads for further cost and demand reduction. An adaptive MFO algorithm was used in [27] where the authors minimised energy costs and peak demand by considering the optimum operation of controllable loads through load shifting. The authors of [34] investigated the SCO for minimising electricity bills and PAR, while ensuring customer comfort, considering different pricing models, such as time-of-use, real-time and critical-peak pricing. Though air conditioners were considered in this research, the power consumption was assumed to be a fixed value and temperature dependencies were taken into account. A comparative analysis among the different optimisation algorithms for building energy management problems was reported in [35], considering time-of-use tariffs. Although the authors considered detailed models for air conditioners, the impact of renewable generation resources was incorporated in the optimisation model. Moreover, the cited research papers did not consider the impact of pre-cooling on the optimum energy management for air conditioners.

Existing demand management solutions available in the market often rely on DLC schemes for customer appliances, such as hot water systems, pool pumps and air conditioners [36]. For example, Energex in Queensland developed and implemented residential air conditioning demand management through the PeakSmart program to address the growing use of air conditioners (ACs), which contribute significantly to peak loads despite being used for relatively short periods of time. This allowed AC manufacturers to develop and commercialize demand response enabling devices (DRED receivers) which can be activated by Energex’s ripple load control system when the electricity network reaches peak demand [37]. However, these programs violate user comfort requirements to reduce peak loads as they are utility controlled and, as a result, there can be low uptake by consumers [38].

Effective demand management solutions for heating and cooling appliances need to address the thermal comfort considerations of end-users. One of the widely used standards for designing and building air conditioner systems is the ANSI/ASHRAE (American Society of Heating, Refrigerating and Air-Conditioning Engineers) Standard 55 which takes into consideration all factors, including temperature, humidity, clothing, air speed, and metabolic rate, among others [38]. The standard uses the predicted mean vote (PMV) model proposed by Fanger, a comprehensive 7-point index which defines comfort as thermal sensations where the neutral temperature is 0 and the range of comfort is between −0.5 to +0.5 [39]. Fanger also considered the correlation between the predicted percentage of dissatisfaction (PPD) with PMV.

Several studies have considered optimising the thermal setting of air-conditioners in commercial buildings. Hsiung et al. proposed the use of PMV as a more accurate representation of human feelings, and particle swarm optimization to find the optimal trade-off between thermal comfort and power consumption in office buildings [2]. The authors of [40] used a binary search algorithm to minimise the aggregate demand and design optimum HVAC control strategies, while taking into account customer comfort. The impact of different thermostat setback scenarios on energy consumption savings was evaluated in [41], but such solutions are deterministic and cannot dynamically adapt to varying customer comfort levels. The authors in [42] minimised the peak and aggregate power demand of the HVAC systems, as well as the difference between actual and preferred temperature set-points for commercial buildings with multiple HVAC zones. Other research papers have considered optimum pre-cooling for commercial buildings. For example, the authors of [43] optimised the pre-cooling strategy in a day-ahead manner, using a thermal model to minimise the electricity cost for a commercial building, while maintaining customer comfort. The optimisation strategy was able to achieve savings of up to 35% in peak demand and 34% in cost by pre-cooling before the start of business hours [43]. The solution has been integrated with an existing building energy management system in Australia through an IoT platform [44]. The authors of [45] considered optimum pre-cooling to minimise voltage violation problems in a low voltage distribution network by shifting the air conditioning load to off-peak hours. It was concluded by the authors of [46] that the advantages of pre-cooling often rely on thermal comfort perceptions and the capacity of the air conditioning units. However, the aforementioned research papers assume that users can respond flexibly to changes in temperature within a given range. In addition, they do not consider that integration of renewable generation in the optimisation framework can further enhance the benefits of pre-cooling.

The key research papers that consider optimum energy management for heating/cooling loads are summarised in Table 1. The table demonstrates that very few research papers have considered pre-cooling for residential buildings and that none of the papers integrated optimum pre-cooling with the utilisation of renewable generation, representing a research gap in the optimum energy management domain.

### 1.2. Research Gaps

The research papers discussed in the previous subsection focus mainly on reducing the energy cost by optimising the operation of air conditioners. Though many of these studies have considered customer comfort, they assume that users can respond flexibly to changes in their preferred temperature. However, there are user groups, such as elderly people and young children, who are vulnerable to changing temperatures. Very recently, pre-cooling technologies have been implemented while satisfying customer comfort to reduce stress on the distribution network. However, the fact that proper utilisation of renewable energy in determining the optimum pre-cooling period can further reduce energy consumption and cost has not received much attention. Given that a significant percentage of residential users have now installed rooftop solar panels, it is essential that optimum pre-cooling strategies are developed by harnessing the joint benefits of renewable generation and lower price periods. In this regard, we have developed an approach for optimum pre-cooling in IoT-enabled smart homes by considering both these factors. Moreover, we consider a fixed temperature as the comfortable temperature (which can be different for different houses or different set-points at different days for the same user) so that users’ comfort level is not sacrificed. To the best of our knowledge, the existing research has not considered such an approach for optimum pre-cooling.

### 1.3. Contributions and Paper Organisation

Based on the research gaps identified, this paper makes the following contributions:

- We develop a multi-stage approach to optimise pre-cooling for air conditioners (AC) in IoT-enabled smart homes which can achieve desired customer set-points while minimising energy costs by shifting the air conditioning load to renewable rich- or low-price hours. The first stage involves optimising the utilisation of renewable generation and can reduce overall electricity consumption. The second stage maximises the use of low-price time periods for pre-cooling the AC and can reduce electricity cost.
- The proposed approach is implemented to investigate the electricity consumption and cost savings using a real world residential energy consumption and generation dataset by considering pre-cooling for summer days during 2012–2013. Given that the impact of structural parameters, such as thermal resistance, is significant for the effectiveness of pre-cooling, the performance of the proposed approach under different thermal resistance settings is also considered.
- The analysis demonstrates that cost savings of at least 15% are achievable when either the daily peak or daily average temperature is the highest. The savings decrease to 6% when the thermal resistance drops by 50%, indicating that the proposed approach is more beneficial for smart homes with good insulation levels. Thus the proposed pre-cooling strategy can be integrated with thermal insulation upgrade initiatives to improve the energy efficiency of residential buildings.

The paper is organised in the following manner: Section 2 describes the energy consumption model of the AC and explains the optimisation problem considered for its pre-cooling. The optimum pre-cooling approach, considering generation and time of use optimisation, is discussed in Section 3. Section 4 illustrates the simulation results on cost savings resulting from the proposed approach. Section 5 concludes the paper.

## 2. Optimum Energy Management for the Ac

The heat gain in a house is closely related to the difference between the indoor and outdoor temperature and the equivalent thermal resistance of the house. Thus, in this section, we first describe a model to define the correlations among the energy consumption of an air conditioner, the indoor and outdoor temperature and the thermal resistance of the house. The energy consumption of the AC at a certain time instant can be represented as a function of the previous instant’s room temperature, the air temperature and the current room temperature, in the following manner [19]:

(1) $$P_{\mathrm{AC},t}=\frac{T_{t-1}^{r}\left(1-\frac{\Delta T}{1000\times M_{a}C_{a}R_{th}}\right)+T_{t-1}^{a}\times \frac{\Delta T}{1000\times M_{a}C_{a}R_{th}}-T_{t}^{r}}{\frac{\mathrm{COP}\times \Delta T}{0.000277\times M_{a}C_{a}}}$$

The above equation shows that, if the room temperature at the previous time instant can be set equal to or slightly lower than the current (desired) room temperature, the energy consumption of the AC can be significantly reduced (even to zero). This clearly indicates that turning on the AC at the set-point or lower temperature beforehand can reduce energy consumption of the AC, especially during peak time. For example, to achieve a temperature of 21 C at 5 p.m., the AC can be turned on at 18 C at 3 p.m. and turned of at 4 p.m. We utilise this fact to optimise the energy consumption of the AC.

Next, we discuss our approach to minimising energy consumption of the AC through pre-cooling. The pre-cooling can be optimised to achieve two goals: (i) reduced electricity consumption (through the use of solar energy), (ii) reduced electricity bills (through the use of a ToU tariff). In both cases, the energy consumption during peak hours is reduced and, as a result, the pre-cooling of the AC can provide a demand response to mitigate blackouts during critical peak demand events. Thus, the optimisation problem for scheduling pre-cooling can be represented as:

(2) $$\min_{v_{t}^{\mathrm{AC}}}\sum_{t=t_{\mathrm{start}}}^{t=t_{\mathrm{end}}}\left(v_{t}^{\mathrm{AC}}P_{\mathrm{AC},t}-P_{\mathrm{excess},t}\right)C_{\mathrm{ToU},t}$$

where $P_{\mathrm{excess},t}$ represents the energy excess available from renewable generation and is given by:

(3) $$P_{\mathrm{excess},t}={\left(P_{G,t}-P_{D,t}\right)}^{+}$$

Note that, in this study, $P_{G,t}$ is assumed to be from rooftop solar generation as we consider residential energy management. However, other types of renewable generation can also be considered in a similar manner and the proposed approach will be equally applicable. Since this study focuses on matching the available renewable generation with the air conditioner demand through optimisation, the approach does not assume any specific models for renewable generation, so, detailed modeling of renewable generation resources is beyond the scope of this paper.

The term $P_{\mathrm{AC},t}$ in (2) is represented by (1). The room temperature needs to be maintained at $T_{\mathrm{SP}}$ from times when the customer wants to achieve the desired set-point temperature until the $t_{\mathrm{end}}^{th}$ instant. Thus,

(4) $$T_{i}^{r}=T_{\mathrm{SP}}\;\forall i\in \left[t_{\mathrm{SP}},t_{\mathrm{end}}\right]$$

Note that thermal comfort is indicated by whether the room temperature can be maintained at the desired set point or not. To achieve the desired temperature at $t=t_{\mathrm{end}}$, the room temperature at the previous time instants needs to be maintained as:

(5) $$T_{t-1}^{r}=\frac{T_{t}^{r}-T_{t-1}^{a}\times \frac{\Delta T}{1000\times M_{a}C_{a}R_{th}}+v_{t-1}^{\mathrm{AC}}\times \frac{\mathrm{COP}\times P_{\mathrm{AC},t}\Delta T}{0.000277\times M_{a}C_{a}}}{1-\frac{\Delta T}{1000M_{a}C_{a}R_{th}}}$$

Note that, when the renewable generation is not sufficient to meet the energy demand, the optimisation solution in (2) aims to minimise the electricity cost based on the ToU tariff. On the other hand, if the user has a flat rate tariff, the solution aims to maximise the usage of renewable generation for pre-cooling the AC. We outline our solutions approach in the next section.

## 3. Optimum Scheduling of Pre-Cooling

We adopt a multi-stage approach to solve the optimisation problem in (2) in a day-ahead manner. The two stages include: (i) Stage 1: *Generation Optimisation* and (ii) Stage 2: *ToU Optimisation*. The following subsections elaborate the objective functions as well as constraints associated with each stage.

### 3.1. Stage 1: *Generation Optimisation*

In this stage, the use of excess energy from renewable generation is optimised to reduce energy consumption from the utility grid. Thus the optimisation problem at Stage 1 can be represented as:

(6) $$\begin{array}{cc} \mathrm{Objective}\mathrm{function}:\;\min_{v_{t}^{\mathrm{AC}}} & \sum_{t=t_{\mathrm{start}}}^{t=t_{\mathrm{end}}}\left(v_{t}^{\mathrm{AC}}P_{\mathrm{AC},t}-P_{\mathrm{excess},t}\right)\\ \mathrm{subject}\mathrm{to} & \\ \mathrm{Constraints}:\;P_{\mathrm{AC},t} & \leq P_{\mathrm{excess},t},\\ P_{\mathrm{AC},t} & =\frac{T_{t-1}^{r}\left(1-\frac{\Delta T}{1000\times M_{a}C_{a}R_{th}}\right)+T_{t-1}^{a}\times \frac{\Delta T}{1000\times M_{a}C_{a}R_{th}}-T_{t}^{r}}{\frac{\mathrm{COP}\times \Delta T}{0.000277\times M_{a}C_{a}}},\\ T_{i}^{r} & =T_{\mathrm{SP}}\;\forall i\in \left[t_{\mathrm{SP}},t_{\mathrm{end}}\right],\\ T_{t-1}^{r} & =\frac{T_{t}^{r}-T_{t-1}^{a}\times \frac{\Delta T}{1000\times M_{a}C_{a}R_{th}}+v_{t-1}^{\mathrm{AC}}\times \frac{\mathrm{COP}\times P_{\mathrm{AC},t}\Delta T}{0.000277\times M_{a}C_{a}}}{1-\frac{\Delta T}{1000M_{a}C_{a}R_{th}}} \end{array}$$

Here, the objective function aims to minimise the difference between total AC power consumption and the total excess solar generation over the operating time period. The first constraint ensures that the AC power consumption does not exceed the solar generation. The second constraint is used to calculate how much power consumption is required by the AC at time *t* so that the set-point temperature can be maintained throughout the time period $\left[t_{\mathrm{SP}},t_{\mathrm{end}}\right]$. The third constraint dictates the temperature set-point and the required time interval over which this temperature should be maintained. This ensures that thermal comfort is maintained throughout the time interval suggested by the user. The last constraint is used to calculate the room temperature at time t−1 so that the desired temperature can be achieved at time *t*. The room temperature at time *t* is calculated based on the air temperature at time t−1 for any time interval before the AC is on. On the other hand, the AC power consumption is calculated backwards in time, that is, how much power is consumed at time $t_{\mathrm{SP}-1}$ to achieve the set-point temperature at time $t_{\mathrm{SP}}$.

After solving the *generation optimisation* problem, the residual energy consumption requirement of the AC to achieve the desired set-point temperature is obtained. If the residual energy consumption is more than zero, then Stage 2 optimisation is performed as described in the following subsection.

### 3.2. Stage 2: *ToU Optimisation*

In this stage, the electricity consumption is optimised to reduce the consumption during peak (high price) hours. Thus the optimisation problem at Stage 2 can be represented as:

(7) $$\begin{array}{cc} \mathrm{Objective}\mathrm{function}:\;\min_{v_{t}^{\mathrm{AC}}} & \sum_{t=t_{\mathrm{start}}}^{t=t_{\mathrm{end}}}\left(v_{t}^{\mathrm{AC}}P_{\mathrm{residual},t}\right)C_{\mathrm{ToU},t}\\ \mathrm{subject}\mathrm{to} & \\ \mathrm{Constraints}:\;P_{\mathrm{AC},t} & =\frac{T_{t-1}^{r}\left(1-\frac{\Delta T}{1000\times M_{a}C_{a}R_{th}}\right)+T_{t-1}^{a}\times \frac{\Delta T}{1000\times M_{a}C_{a}R_{th}}-T_{t}^{r}}{\frac{\mathrm{COP}\times \Delta T}{0.000277\times M_{a}C_{a}}},\\ T_{i}^{r} & =T_{\mathrm{SP}}\;\forall i\in \left[t_{\mathrm{SP}},t_{\mathrm{end}}\right],\\ T_{t-1}^{r} & =\frac{T_{t}^{r}-T_{t-1}^{a}\times \frac{\Delta T}{1000\times M_{a}C_{a}R_{th}}+v_{t-1}^{\mathrm{AC}}\times \frac{\mathrm{COP}\times P_{\mathrm{AC},t}\Delta T}{0.000277\times M_{a}C_{a}}}{1-\frac{\Delta T}{1000M_{a}C_{a}R_{th}}} \end{array}$$

Here, the objective function minimises the electricity cost by considering the different pricing for peak and off-peak intervals. The three constraints are similar to those in Stage 1, as explained in the previous subsection. The solution approach combining the two optimisation stages is explained below.

### 3.3. Solution Approach

At the beginning, the temperature set-points for the next day are requested from the user. For a specific set-point at $t=t_{\mathrm{end}}$, the previous time instants in the range $\left[t_{\mathrm{start}},t_{\mathrm{end}}-1\right]$ are considered for optimum scheduling of the pre-cooling. That is, the pre-cooling can start from at most $t_{\mathrm{end}}-t_{\mathrm{start}}$ before the desired set-point temperature needs to be achieved.

For each pre-cooling time instant in the range $\left[t_{\mathrm{start}},t_{\mathrm{end}}-1\right]$, the pre-cooling temperature needs to be calculated using (5), which ensures that the desired temperature is achieved at $t=t_{\mathrm{end}}$ and no further cooling is needed. First, the *generation optimisation* stage will be considered for a certain pre-cooling time instant. Here, the available renewable generation is used to achieve the target pre-cooling temperature. For each pre-cooling time instant, the electricity consumption of the AC to achieve the pre-cooling temperature goal is calculated using (1). If the available renewable generation is more than the required $P_{\mathrm{AC}}$, the target pre-cooling temperature is considered to be achieved. If the available renewable generation is not sufficient to meet the pre-cooling temperature goal, the available renewable generation is used for the AC. The energy consumption at each pre-cooling time instant and the associated energy cost is stored as a candidate solution. Next, the *ToU optimisation* stage will be considered for the same pre-cooling time instants. Here, the pre-cooling is scheduled if a low tariff is available for the considered pre-cooling hour. The energy consumption and cost results from the previous stage are updated accordingly. The optimisation problems are solved using an exhaustive search through all possible solutions.

After each stage of optimisation, the room temperatures (or set-points) for the subsequent time instants are updated. Once the energy consumption and energy cost are calculated for all the possible pre-cooling time instants, the energy cost of the solutions is compared with the previous best solution. Initially, the best solution is the energy cost with no pre-cooling. If pre-cooling improves the energy cost, the temperature values are updated based on the solutions. The process is repeated for each time instant for which the temperature set-point is defined by the user.

**Figure 1:**
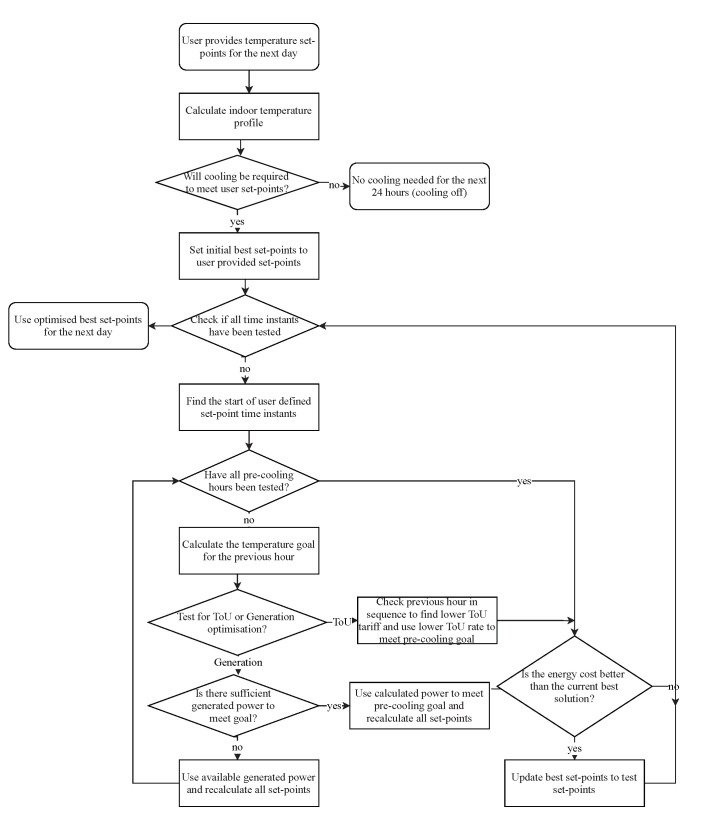
Solution approach for optimum scheduling of the AC pre-cooling.

The approach is illustrated in the flowchart in Figure 1.

## 4. Results

In this section, we evaluate the implementation of the proposed optimum scheduling approach. The following subsection provides details of the case study we considered in this paper.

### 4.1. Case Study Description

We used the Ausgrid solar home electricity dataset which defines the energy consumption and generation values for 300 houses in New South Wales, Australia, at 30 min intervals between 1 July 2012 and 30 June 2013 [47]. The dataset comprises general consumption, controlled load and gross generation data for each of the 300 houses connected to the Ausgrid electricity network. The average solar panel capacity of these houses is 1.68 kW with average daily generation of 5.97 kWh/day and average daily consumption of 17.5 kWh/day. Historical ambient temperature data was collected from the Open Weather Map application programming interface (API) for the same location [48]. We consider a ToU tariff structure, as shown in Table 2.

We consider a set-point temperature of 25 C from 5 p.m. to 9 p.m. for all houses in the dataset. There are 41 days in the dataset when the ambient temperature is high enough to use the AC for cooling. From our analysis, we found that, for 37 days, most of the houses in the dataset achieved improvements in energy consumption and cost when the proposed optimisation scheme was implemented. The houses which did not achieve improvements, had sufficient renewable generation and lower energy demand, which allowed them to achieve the desired set-point temperature without consuming from the utility grid.

**Figure 2:**
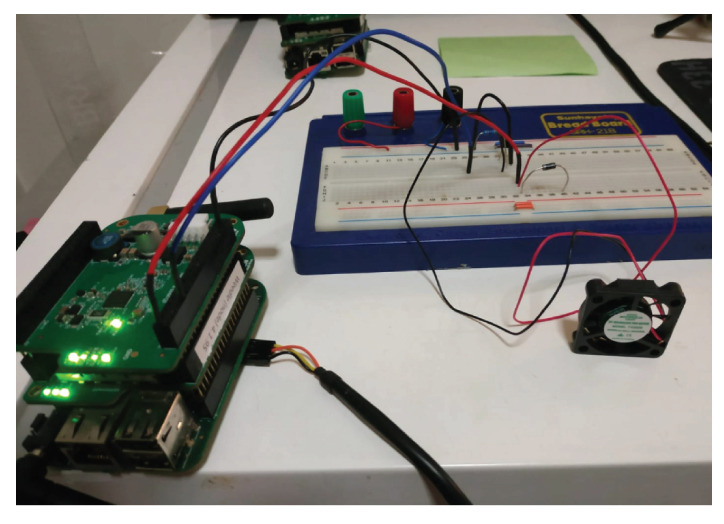
Implementation using Itron Riva board.

We also implemented the algorithm using an Itron Riva board, which is an embedded Linux computer board with memory and storage. The algorithm is written in Python and run in the board using shell scripts. A general purpose input output (GPIO) pin was used to run a small 5V fan during optimal times. The implementation setup is demonstrated in Figure 2.

In this paper, we demonstrate the improvements for a specific house, ID 165, for days with highest average (8 January 2013) and highest peak temperature (18 January 2013). In addition, the impact of thermal resistance on the energy consumption for the AC is investigated. Finally, the reduction in peak kW consumption over the 41 high temperature days during December, 2012 to February, 2013 is also demonstrated. House ID 165 had a solar panel capacity of 1.02 kW. The thermal resistance of the house was considered to be $3.1965\times 10^{-6}$C/kW, unless otherwise specified. In this analysis, we assume $M_{a}$ = 1778.369 kg, $C_{a}$ = 1.01 kJ/kgC., COP = 2 [19].

### 4.2. Highest Average Temperature Day

**Figure 3:**
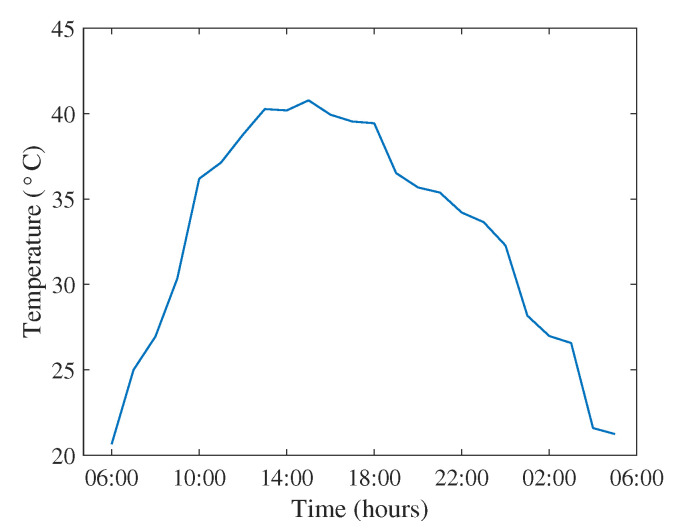
Ambient temperature on 8 January 2013.

**Figure 4:**
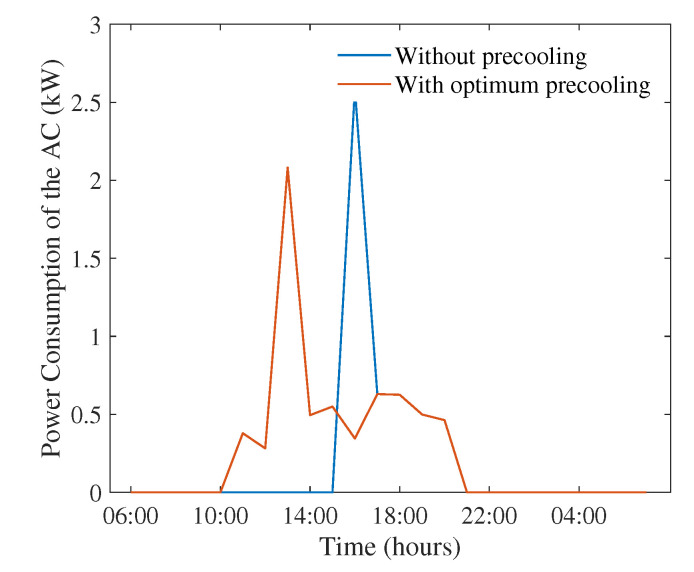
Power consumption of the AC with and without optimum pre-cooling on 8 January 2013.

Based on the temperature data from [48], it was found that the highest average temperature day (when averaged over 24 h) was 8 January 2013. The ambient temperature for this date is shown in Figure 3, where the peak temperature 40.78C occurred at 2 p.m. The impact of the proposed optimisation scheme on the energy consumption of the AC is shown in Figure 4. It can be seen that the proposed optimisation scheme is able to move consumption of the AC from 4 p.m. to 1 p.m., taking advantage of high levels of renewable generation during mid-day, as well as lower tariffs. The peak consumption of the AC is also decreased from 2.5 kW to 2 kW, when the proposed scheme is used.

**Figure 5:**
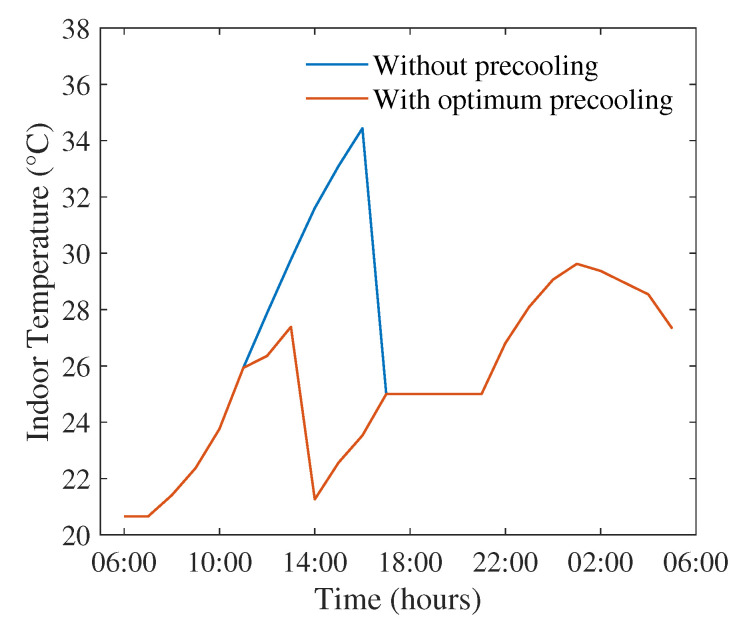
Indoor temperature with and without optimum pre-cooling on 8 January 2013.

The indoor temperature when optimum pre-cooling is applied is shown in Figure 5. Instead of allowing the indoor temperature to increase above the set-point and then operating the AC to cool the house down, the proposed scheme lowers the temperature slightly below the set-point earlier on. At 2 p.m., the ToU pricing changes into peak pricing. Before the price changes into peak pricing, additional cooling is supplied from the grid to reach 21.26 C at 1 p.m. As a result of pre-cooling, no further cooling is needed at 5 p.m. at the start of set-point hours, which is normally the largest expense if pre-cooling is not applied. The proposed strategy ensures that the temperature remains at the set point 25 C from 5 p.m. to 9 p.m., as required by the user, which indicates that the required thermal comfort can be maintained with the proposed pre-cooling strategy. Moreover, when no pre-cooling is applied, the indoor temperature rises to 34 C, which is 27 C when optimum pre-cooling is used. Thus, the proposed strategy can minimise the chances of extremely uncomfortable temperatures throughout the entire day.

### 4.3. Highest Peak Temperature Day

**Figure 6:**
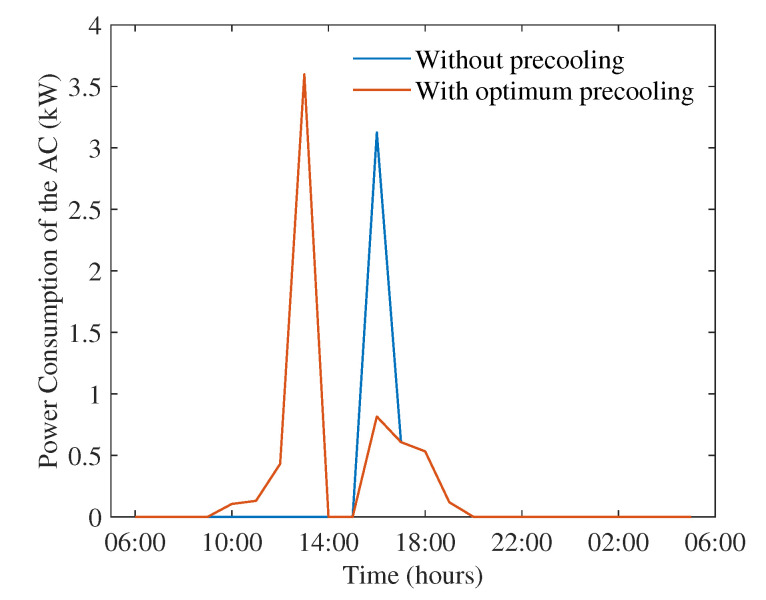
Power consumption of the AC with and without optimum pre-cooling on 18 January 2013.

We also investigated the reduction in energy consumption for the day with the highest peak temperature, which was 18 January 2013. The additional cooling requirements for this date would have been higher as the peak temperature during the day was much higher 44.75C at 2 p.m. The changes in energy consumption of the AC due to the proposed scheme is illustrated in Figure 6. Though the peak consumption of AC increases to 3.5 kW with the proposed scheme from the no pre-cooling case of 3 kW, the peak is shifted to 1 p.m., when sufficient renewable generation is available. The maximum consumption during high price hours is limited to 1 kW.

### 4.4. Impact of Thermal Resistance

**Figure 7:**
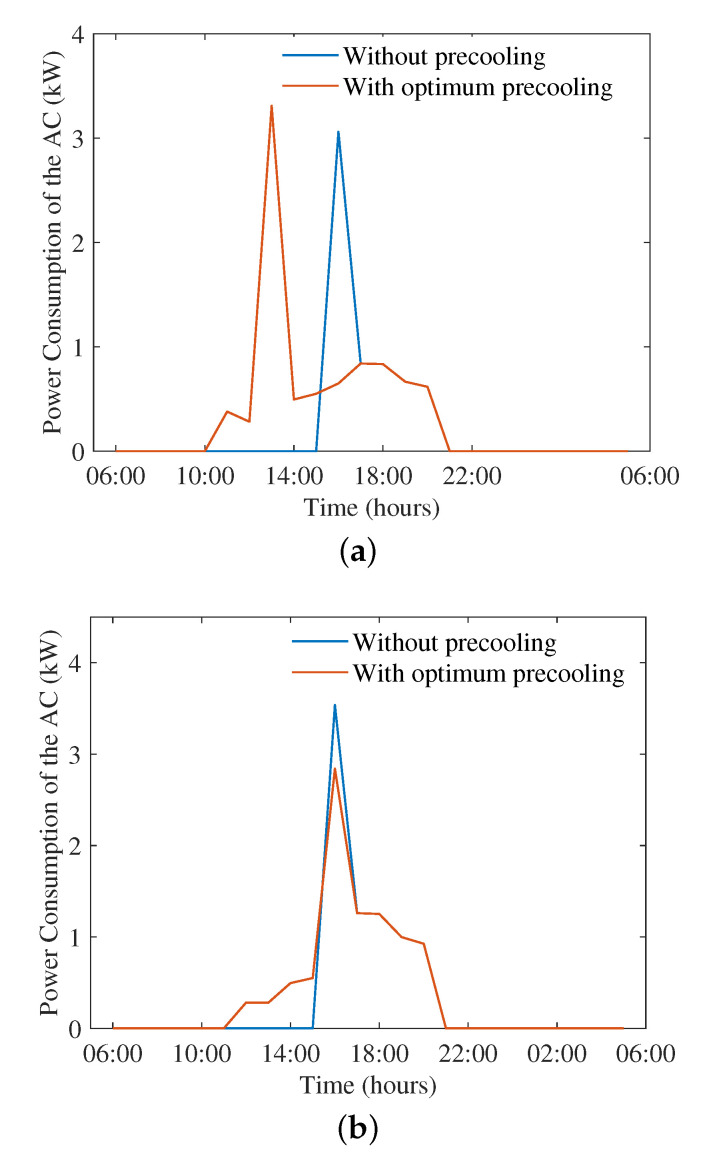
Impact of thermal resistance on the power consumption of the AC with and without optimum pre-cooling. (**a**) Thermal resistance = 2.3974 × 10${}^{-6}$. (**b**) Thermal resistance = 1.5982 × 10${}^{-6}$.

In this section, we consider the reduction in thermal resistance of the house ID 165 by 25% and 50%, and evaluate the performance of the proposed scheme. Figure 7a,b show the impact of the proposed scheme on energy consumption of the AC when thermal resistance was reduced to 2.3974 × 10${}^{-6}$C/kW and 1.5982 × 10${}^{-6}$C/kW. The day considered in this case was the highest average temperature day in the dataset, 8 January 2013. Thermal resistance is an indicator of how easily the heat transfer can take place between the indoor and outdoor environments. A lower thermal resistance value means a faster rate of heat exchange. With a 25% reduction in thermal resistance, the peak energy consumption of the AC increases to 3.3 kW with optimum pre-cooling from 3 kW with no pre-cooling.

When the thermal resistance is reduced by 50%, the heat exchange between the indoor and outdoor environments happens at a faster rate. The earliest hours of pre-cooling before 12 p.m. are no longer effective as the cooling benefit is lost due to the fast heat exchange. The peak consumption of the AC decreases slightly from 3.5 kW to 3 kW when the proposed scheme is used. In this case, the savings are achieved by the generation optimisation stage only. The ToU optimization is not useful in the solution as the cost of cooling outweighs the pre-cooling benefits given that the cooling requirements are retained from 5 p.m.

### 4.5. PAR and Cost Savings

**Figure 8:**
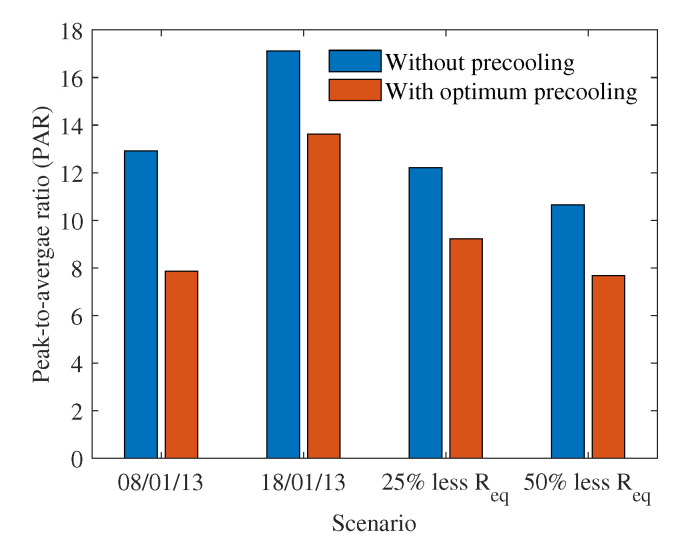
Peak-to-average ratio of house 165 for the different scenarios considered in the case study.

In this subsection, the PAR and electricity cost savings for house 165 under the different scenarios considered in the previous subsections are investigated to understand the impact of pre-cooling. Figure 8 shows the peak-to-average ratio of the electricity consumption of house 165 without and with optimum pre-cooling. The PAR is lower with optimum pre-cooling because the proposed strategy moves electricity demand from peak to off-peak hours providing more solar generation to offset the electricity demand. Moreover, the proposed strategy spreads demand over a greater number of time periods, reducing the sudden high consumption. The highest maximum temperature day had a higher PAR, meaning that the peak consumption was much higher compared to the average consumption for the given thermal resistance. When thermal resistance is less, the benefits of optimum pre-cooling for reducing the PAR are less, as can be seen for the 25% lower thermal resistance. When thermal resistance continues to decrease to 50% less thermal resistance, PAR decreases, meaning that both peak demand and average demand are high and the AC is used for longer time periods.

**Figure 9:**
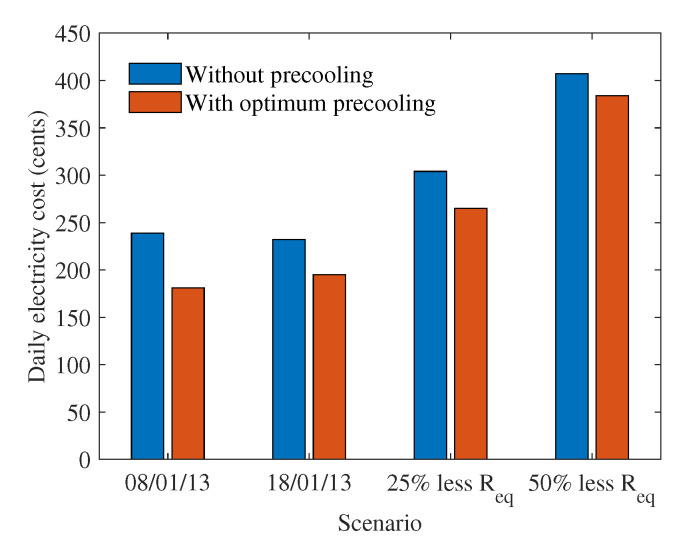
Electricity costs for house 165 for the different scenarios considered in the case study.

Similar trends can be observed from the daily electricity costs shown in Figure 9. Optimum pre-cooling can achieve lower electricity costs for all the scenarios. During the highest average temperature day, the total cost of cooling is reduced by 24.25%, the majority of which comes from the generation optimisation stage. On the other hand, for the highest peak temperature day, the improvement in energy cost is 15.95%, half of which can be attributed to the generation optimisation stage. When no pre-cooling is used and thermal resistance decreases by 25%, the energy cost increases by 27.2% compared to the energy cost with no pre-cooling when thermal resistance is higher. With the proposed scheme, the energy cost can be improved by 12.54% compared to the no pre-cooling case. Note that this improvement is 24.25% when the thermal resistance value is higher. When thermal resistance decreases by 50% and no pre-cooling is used, the energy cost increases by 70.3% compared to the energy cost with no pre-cooling when thermal resistance is 50% higher. With optimum pre-cooling, the energy cost improves by 6%. Thus, the cost savings become smaller when thermal resistance values are less.

### 4.6. Peak Hour Energy Consumption

**Figure 10:**
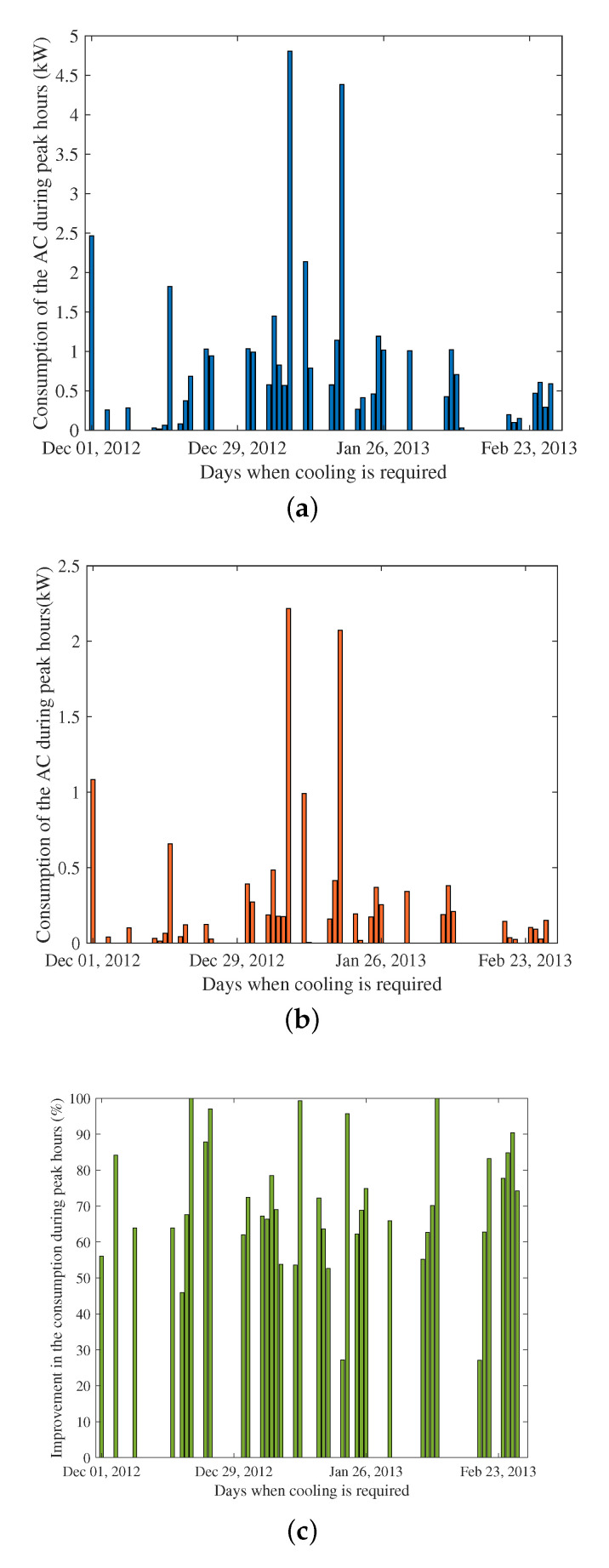
Power consumption of the AC during peak hours with and without optimum pre-cooling. (**a**) No pre-cooling. (**b**) Optimum pre-cooling. (**c**) Improvement.

Here we investigate the daily consumption of the AC during peak hours for House ID 165 over the days when cooling was required and evaluate the savings in daily peak hour consumption with the optimum pre-cooling scheme. Peak hours of use in NSW occur between 2 p.m. and 9 p.m. and the analysis was performed for the energy consumption of the AC during these hours. Figure 10a,b show the daily peak hour consumption of AC without and with pre-cooling, whereas Figure 10c demonstrates the percentage improvement in daily peak hour consumption. The maximum consumption of the AC during peak hours was around 4.8 kW when no pre-cooling was used in January 2013. When optimum pre-cooling is used, the peak hour consumption of the AC decreases to 2.3 kW, which represents a significant improvement. From Figure 10c, it can be seen that the daily peak hour consumption of the AC improves for 38 days over the summer. For three days, there is 100% improvement, indicating that the AC does not consume any energy during peak hours when pre-cooling is used.

## 5. Conclusions

This paper proposes a new approach to optimise the pre-cooling operation for ACs in IoT-enabled smart homes. The approach maximises the utilisation of renewable generation, as well as the benefits of consuming energy at a low price period. The proposed optimum pre-cooling approach was applied to compute the energy cost savings based on renewable generation, weather and energy price data for NSW, Australia. The analysis showed cost savings of 24% and 15% for the day with highest average temperature and highest peak temperature in the 2012–2013 summer period. Moreover, the peak energy consumption of the AC could be reduced for 38 days of the 41 days when cooling was required. Future work will focus on extending the analysis to accommodate other types of thermostatically controlled loads, such as electric heaters and water heaters.

## Figures and Tables

**Table 1:** Summary of key research papers on optimum energy management for heating/cooling appliances.

References	Renewable Energy	Time Varying Pricing	Pre-Cooling
[30]	×	×	×
[34]	×	✓	×
[35]	×	✓	×
[2]	×	×	×
[40]	×	×	×
[42]	×	×	×
[43]	×	✓	✓
[44]	×	✓	✓
[45]	×	✓	✓
[46]	×	×	✓
Proposed study	✓	✓	✓

**Table 2:** ToU residential tariff for Ausgrid distribution zone [49].

Time	Rate (cents/kWh)
7 a.m.–2 p.m.	23.89
2 p.m.–8 p.m.	53.01
8 p.m.–10 p.m.	23.89
10 p.m.–7 a.m.	14.42

## Data Availability

Not applicable.

## Nomenclature

$P_{\mathrm{AC},t}$	Power consumption of the AC at time *t*
$T_{t-1}^{r}$	Room temperature at time t−1
ΔT	Time duration
$M_{a}$	Mass of the air
$C_{a}$	Thermal capacity of air
$R_{th}$	Equivalent thermal resistance
$T_{t-1}^{a}$	Ambient temperature at time t−1
$T_{t}^{r}$	Room temperature at time *t*
COP	Coefficient of performance
$v_{t}^{\mathrm{AC}}$	Binary variable, 1 means AC is operating, 0 means AC is not operating
$t_{\mathrm{start}}$	Time when AC starts to operate
$t_{\mathrm{end}}$	Time when AC finishes operation
$P_{\mathrm{excess},t}$	Energy excess available from renewable generation at time *t*
$C_{\mathrm{ToU},t}$	Time-of-use tariff at time *t*
$P_{G,t}$	Renewable generation at time *t*
$P_{D,t}$	Energy demand at time *t*
$T_{\mathrm{SP}}$	Set-point temperature
$t_{\mathrm{SP}}$	The first instant when customer wants to achieve the set-point temperature
$P_{\mathrm{residual},t}$	The residual energy consumption of the AC at time *t* which could not be
	satisfied from excess generation
